# Medical informatics and climate change: a framework for modeling green healthcare solutions

**DOI:** 10.1093/jamia/ocac182

**Published:** 2022-10-11

**Authors:** Marieke E Sijm-Eeken, Welmoed Arkenaar, Monique W Jaspers, Linda W Peute

**Affiliations:** Department of Medical Informatics, Amsterdam UMC location University of Amsterdam, Centre for Sustainable Healthcare, Amsterdam Public Health Institute, Amsterdam, The Netherlands; Department of Medical Informatics, Amsterdam UMC location University of Amsterdam, Amsterdam, The Netherlands; Department of Medical Informatics, Amsterdam UMC location University of Amsterdam, Center for Human Factors Engineering of Health Information Technology, Amsterdam Public Health research institute, Amsterdam, The Netherlands; Department of Medical Informatics, Amsterdam UMC location University of Amsterdam, Center for Human Factors Engineering of Health Information Technology, Amsterdam Public Health research institute, Amsterdam, The Netherlands

**Keywords:** environmental impacts, medical informatics, climate change, theoretical models, healthcare reform

## Abstract

**Objective:**

The aim of this study was to develop a theory-based framework to enhance and accelerate development, selection, and implementation of solutions mitigating the climate impact of healthcare organizations.

**Materials and Methods:**

Existing frameworks were combined to develop the Green-MIssion (**M**edical **I**nformatic**s S**olut**ion**s) framework. It was further developed and refined by mapping solutions from project plans and reviewing it with an expert panel.

**Results:**

The framework classifies solutions into three categories: (1) monitor and measure environmental impact of a healthcare setting; (2) help create and increase awareness among employees and patients; and (3) interventions to reduce environmental impacts.

**Discussion and Conclusion:**

The framework combines concepts from healthcare information technology and environmental sciences and can be used to structure green medical informatics solutions for different healthcare settings. Furthermore, research should evaluate its application for measuring and assessing the impact of green medical informatics solutions on environmental sustainability and climate resilience.

## BACKGROUND AND SIGNIFICANCE

The healthcare sector is responsible for producing 4.4%–4.9% of global net carbon dioxide emissions.^1^^,^^2^ In developed countries like the United States, the carbon emissions of health care are estimated to be much higher.^2^^,^^3^ Health care is also responsible for other factors that impact the environment, like the disposal of pharmaceuticals in waste water (in healthcare facilities and patient homes), fresh water usage and producing waste that is processed as landfill or incinerated.^4–6^

The healthcare sector is also impacted by the effects of climate change, for example through the increasing number of people affected by storms, flooding, droughts, wildfires, and the growing number of insect-borne diseases.^7^ Between 2030 and 2050, the World Health Organization estimates that climate change will cause 250 000 extra deaths per year due to hunger, malaria, diarrhea, and heat stress.^8^ Striving to improve public health and prevent and treat health problems, management, policymakers, and healthcare practitioners aim to reduce emissions and increase resilience to climate change effects. We use the term “resilience” to describe a healthcare organization's ability to continue providing care while adapting and transforming to meet the challenges posed by climate change.

The World Health Organization provides a roadmap for decarbonizing health care to better comply with the Paris Agreement internationally.^9^ On a national level, communities should share best practices and generate ideas for reducing healthcare emissions. Even though top-down advice and recommendations are important, changes in the daily operations of healthcare organizations and care networks are also required to achieve reductions in emissions. Information technology (IT) provides opportunities and solutions to support such changes.^10^^,^^11^

Even though its potential is recognized, the application of IT in health care for mitigating environmental impact or “*green medical informatics solutions*” has rarely been studied. An exception is the use of telemedicine where multiple studies confirm that telemedicine reduces the amount of travel-related carbon emissions.^12^ Other examples of green medical informatics solutions include cloud solutions, Lean Six Sigma, Green IT and Data Science to measure, analyze, and improve the environmental footprint of the sector.^13^^,^^14^

The limited amount of research and best practice sharing on green medical informatics solutions can be explained by the lack of professionals with knowledge about both the medical informatics and environmental sciences domains. Most medical informatics professionals lack expertise in the environmental impacts of healthcare and environmental experts lack knowledge about medical informatics. Hence, the enormous potential of medical informatics to speed progress in climate-related development and research remains untapped.

## OBJECTIVE

This article describes the iterative development and first version of the Green-MIssion (**M**edical **In**formatic**s S**olut**ion**s) framework: a framework for structuring medical informatics solutions and their influence on and relationship with the environmental impacts of health care. The framework aims to provide healthcare practitioners, researchers, and decision-makers a means to initiate, structure, and share medical informatics solutions and their environmental impact. Its overarching objective is to aid in the design and targeting of interventions for climate change adaptation and mitigation.

## MATERIALS AND METHODS

The Green-MIssion framework was developed in five steps (see Supplementary Appendix SA for details). First, framework requirements were identified in a case study with experienced healthcare professionals. Second, existing frameworks in healthcare interventions, IT solutions, and environmental impacts were identified from the literature and compared with the requirements. Third, the comparison was used to generate an initial version of the framework for modeling medical informatics solutions and their interaction with the environment. The framework was refined by mapping solutions from project plans to the framework (Supplementary Appendix SB). Finally, the framework was adjusted based on feedback from an expert panel.

## RESULTS

Six requirements for a framework for modeling and describing medical informatics solutions and their environmental impact were identified by conducting interviews. All participants agreed that the framework should trigger idea generation on new/extended solutions and facilitate sharing of solution designs within and outside organizational boundaries. The framework should also model the solutions architecture as well as its relation to environmental impacts. Additional requirements included the framework’s capacity to model the complete set of changed structures in a healthcare setting (including technical, functional, process, and governance items) and the future state (excluding the migration path from the current to the future state).

Based on the comparison of existing frameworks with these requirements (Table 1), the 3LGM model^15^ and the WHO framework^9^ were selected to contribute to an initial version of the Green-MIssion framework. Elements included IT Solution architecture (expanded with Governance), Climate Resilience and Environmental impacts.

**Table 1.** Requirements for a framework for modeling medical informatics solutions and their impact on the environment compared to existing frameworks

	Requirements								
	Model solution architecture	Model environmental impact of solution	Model complete set of changed structures healthcare setting				Describe future state	Trigger idea generation	Facilitate sharing of solution
			Technical	Functional	Process	Governance			
3LGM from Winter et al^15^	Yes	No	Yes	Yes	Yes	No	Yes	?	Yes
WHO^9^	No	Yes	No	No	No	No	Yes	Yes	?
Mintzberg^16^	No	No	No	No	Yes	Yes	Yes	No	No
OECD^17^	No	No	No	No	Yes	Yes	Yes	No	No
DPSIR from Ness et al^18^	No	Yes	No	No	No	No	No	Yes	Yes

Yes: this requirement is met. No: this requirement is not met. ?: unclear if the framework/model meets this requirement.

Nineteen project plans (Supplementary Appendix SB) developed for a large academic hospital in the Netherlands to make environmental improvements were then mapped to the first version of the framework. During the mapping, the research team added “Solution type” as an element to distinguish the primary aim of a solution either as directly mitigating environmental impacts or as measuring and understanding environmental impact. To facilitate the sharing of solutions, “type of healthcare facility” was also added as an element.

### Green-MIssion framework

**Figure 1.**
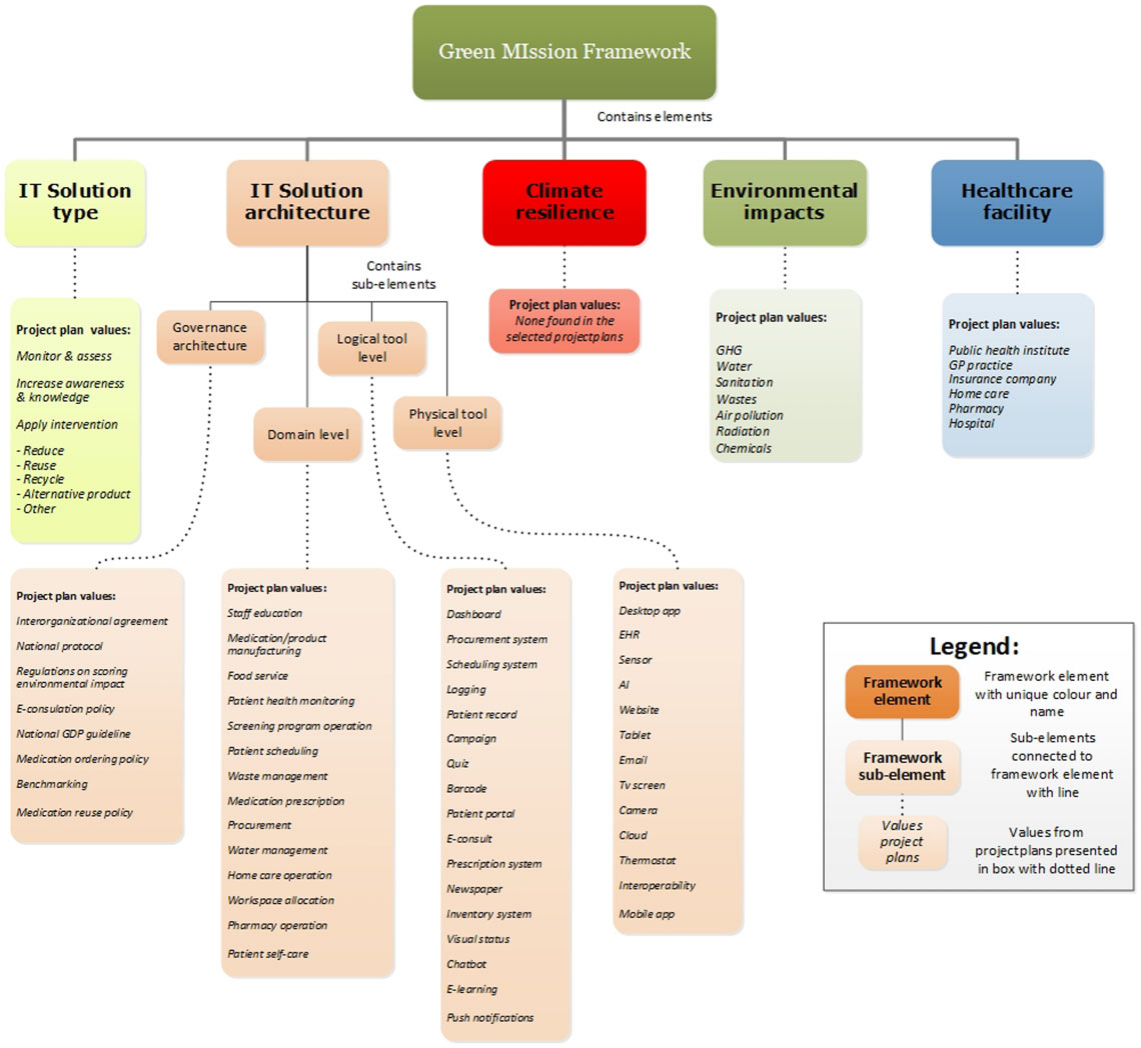
Values from project plans for each framework (sub-)element.

Figure 1 presents an overview of values described for each element of the first version of the framework based on the project plans. No project plans addressed climate resilience. A description of individual solutions with corresponding values for framework elements is presented in Supplementary Appendix SC.

Examples of solutions related to *monitoring and assessing* environment and climate problems and solutions included dashboards presenting environmental impact precursors, such as distance traveled or waste volume; and a report with environment-related disease incidence (eg, heat stroke or respiratory diseases linked to poor air quality). Solutions focusing on *awareness and knowledge* included warnings for potential unnecessary environmental impact (eg, when describing large amounts of medication, when ordering disposable products) and information systems showing or sorting the environmental impact of treatment options. Solutions in the *interventions* category aimed to reduce the use of products, increase the re-use of products, or increase the recycling of materials. Examples include using telemedicine to reduce carbon emissions from travel, realizing paperless processes through electronic information systems, and enhancing the sharing of medication data between pharmacies and home-care to reduce medication waste.

**Figure 2.**
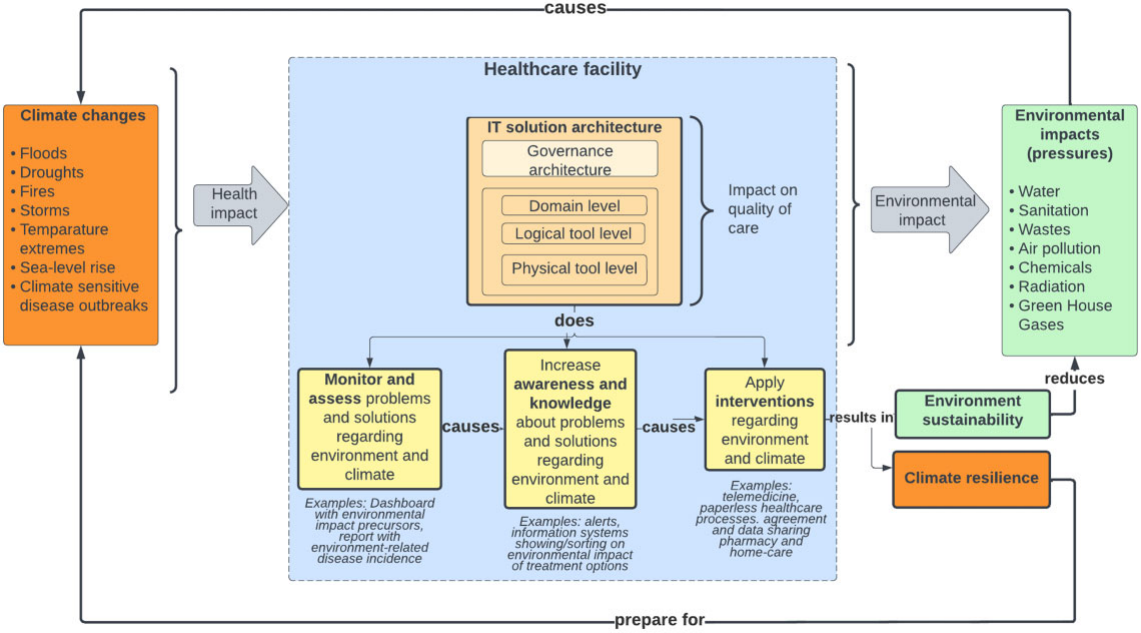
The Green-MIssion (**M**edical **I**nformatic**s S**olut**ion**s) framework.

Reviewing the framework with an expert panel resulted in confirming elements of the framework and in clarifying relationships between elements. Quality of care was added to the framework to highlight the important relationship between medical informatics solutions and healthcare quality improvement. Figure 2 presents the resulting framework.

The final Green-MIssion framework models the different climate events that may impact public health and their environmental impact on healthcare facilities. The center of the framework is the healthcare facility organization type (hospital, pharmacy, etc.). Within the facility, IT solutions can help prepare for changed demand resulting from climate change (“climate resilience” solutions) and/or impact the environmental sustainability of the facility. An IT solution architecture can be described by the levels of governance, domain (enterprise functions or processes and entity types), logical tools (application components) and physical tools (physical data processing components). The *Solution type* classifies green medical informatics solutions into three types based on their aims: *apply interventions* to reduce environmental impact or improve resilience, *increase knowledge and awareness* about problems and solutions, or *monitor and assess* environment and climate problems and solutions in healthcare organizations. To describe interorganizational solutions (eg, changing care pathways based on medication usage and information collected in the home environment to feed electronic health records), the framework can describe the solution from the perspective of each of the healthcare facilities involved.

## DISCUSSION

The proposed framework combines medical informatics solutions and environmental impacts. It offers guidance to professionals involved in information system design, development, or implementation to actively contribute to meeting the global challenges related to climate change. For this purpose, the three main categories of solutions that form the heart of the framework are a pragmatic starting point to consider how information systems can be adjusted or designed to: (1) measure and monitor problems and solutions regarding the environment and climate, (2) increase awareness on these problems and solutions, and (3) implement interventions to mitigate problems and impacts. For example, by considering these three types of solutions a team of healthcare professionals can brainstorm on possible solutions in a structured way. The framework offers guidance for sharing best practices, generating idea, and modeling solutions based on their implications to the environment and their broader context in terms of quality of care, for example, identifying in which circumstance (IT solution architecture and healthcare facility) telemedicine is successful.

In this study, the framework was used to model new green medical informatics solutions specifically designed to address environmental impacts. However, solutions can bring environmental benefits even if this was not the primary goal. An example is the use of teleconsultation solutions during COVID-19 pandemic, intended to continue delivering care while limiting physical contact and limit new infections with the virus but also reduced travel and carbon emissions.^19^ At the same time, changes to information systems in health care might worsen the environmental impact of health care. An example is the introduction of a new application to improve data exchange that increases energy usage. Therefore, we should model and evaluate environmental impacts of all new IT solutions in health care in favor of the environment. Investigating how effective the framework supports such modeling and evaluation is a topic for future research. The power of the Green-MIssion framework lies in its ability to support ideation, exploration, and sharing best practices related to green medical informatics solutions without needing a thorough understanding of environmental sciences.

### Limitations

A specific selection of existing models was used as a foundation for the framework. Other frameworks exist that could potentially provide additional relevant concepts including models reflecting organizational structure and sociotechnical models.^20^

The framework does not include elements to describe the environmental impact of the solutions themselves. In the physical tool and domain layers of solution architecture, choices in software development approaches can influence the energy consumed by software solutions, for example, through the use of energy-efficient programming techniques.^21^ Power demands of software relate to the coding efficiency but also to the platform for which the code is developed, hosting and maintenance materials, and usability characteristics. The complete life cycle from “cradle to grave” of healthcare solutions should be considered when assessing environmental impacts.^14^

In its current form, the framework is not intended to assess and compare the environmental impact of different types of solutions. Further development may focus on expanding the framework with knowledge from Health Technology Assessment (HTA).^22^ For this, insights are also needed into measurable indicators of a healthcare service’s environmental impact. Thus, outcomes of lifecycle assessments and findings from usability or HTA studies can aid in the design, comparison, and evaluation of medical informatics solutions.

The framework can also be improved through further evaluation and testing. In this study, testing was based on solutions described in project plans rather than real-life implementations. Because project plans often prove to be too optimistic or unrealistic, evaluating the framework by reviewing implementation results can provide valuable insights.^23^ An important first step is to measure the baseline before solutions are implemented.^9^ We are working on establishing a theory-based measurement approach for performing before and after implementation measurements and adequately evaluate solution implementations.

A more extensive review of the framework may provide better insights in how well it meets our stated requirements. For example, its usefulness in sharing solutions across organizations could be evaluated by a qualitative study with a sufficient number of participants by using standardized questionnaires. Another gap is that the framework has not been tested on solutions addressing climate resilience. Although little has been published about medical informatics solutions focusing on climate resilience, many solutions for increasing climate resilience enhance facility management.^24^ It is certainly possible that medical informatics can contribute, for example, through the use of AI solutions.^25^

## CONCLUSION

A framework developed by combining theory from hospital information management and environmental sciences supports modeling of green medical informatics solutions and their relationship to environmental impacts. The framework can aid idea generation on solutions and first results indicate it is capable of supporting the sharing of solution designs within and outside organizational boundaries.

## FUNDING

This research received no specific grant from any funding agency in the public, commercial, or not-for-profit sectors.

## AUTHOR CONTRIBUTION

All individuals who meet the requirements for authorship are identified as authors, and each author attests that they contributed sufficiently to the work to assume responsibility for its content, including conception, design, analysis, writing, or manuscript revision.

All authors contributed to the conception and design of the study. The acquisition of data was done by MSE, and the analysis and interpretation of data was done by MSE and WA.

Authors MSE and WA substantially contributed to the drafting of the article and LWP and MWJ contributed to revising the manuscript critically.

All authors gave final approval of the version of the manuscript to be published. All authors agree to be accountable for all aspects of the work.

## SUPPLEMENTARY MATERIAL

Supplementary material is available at *Journal of the American Medical Informatics Association* online.

## Supplementary Material

ocac182_Supplementary_Data

## Data Availability

The project plans that support the findings of this study are available on request from the corresponding author, MSE. The project plans are not publicly available due to the project plans containing information that could compromise the privacy of research participants.
