# Supplementary material for: Medical informatics and climate change: a framework for modeling green healthcare solutions
Source: J Am Med Inform Assoc. 2022 Oct 11;29(12):2083–8. doi: 10.1093/jamia/ocac182 (PMC9667184; doi:10.1093/jamia/ocac182)
Supplement: ocac182_Supplementary_Data [file ocac182_supplementary_data.zip › ocac182_Supplementary_Data/Supplementary File Appendix A.docx]

Appendix A. Research protocol

***Framework requirements***

| **Research methodology** | **Description** |
| --- | --- |
| Framework requirements – interview candidate selection and characteristics | Sampling was used for selecting participants for the interviews in this study. Participants were selected based on criteria:  1. *Expertise*: Was involved for more than 2 years in projects together with multiple green teams for implementing green medical informatics solutions  2. *Role*: Is a targeted user of the framework (researcher, policy maker, project manager, ICT consultant, environmental expert) in a healthcare setting. This resulted in four interview candidates (one ICT consultant and three project manager). Interviews were conducted by the first author with undergraduate level expertise in Environmental Sciences to gain insight in the requirements. Open questions were used to explore requirements about the content and usage of a framework for modeling green medical informatics solutions to allow interviewees to express their open opinion. |
| Expert panel member selection and characteristics | Members of the expert panel were selected based on diversity of expertise on medical informatics fields, availability and involvement in sustainable healthcare education, research or projects in a healthcare setting. Four experts were involved in the evaluation of the framework.  Panel members and their characteristics are described in the table below. There is no overlap between participants selected for the interviews in which requirements for the framework were identified and the expert panel members.   \| **Panel member nr.** \| **Role (Organization)** \| **Expertise area** \| \| --- \| --- \| --- \| \| 1 \| Advisor healthcare innovation (National Public Health Institute) \| E-health  Healthcare policy  Healthcare innovation  Green care \| \| 2 \| Lean Six Sigma master black belt (Academic Hospital) \| IT Governance  Healthcare process optimization  Sustainable healthcare projects  Environmental Sciences (undergraduate level) \| \| 3 \| Principle Investigator Medical Informatics (Academic Hospital) \| Usability evaluation and design  Socio-technological interaction \| \| 4 \| Environmental coordinator (Academic Hospital) \| Environmental sciences (Graduate level), environmental impacts & causes \| |
| Selection of project plans | The project plans that provided input for refining the model were identified during a four week medical informatics course on sustainable healthcare, given at the University of Amsterdam in a collaboration between students and Green Teams. Contents and concepts used in healthcare climate/sustainable project plans based on short traineeships within a medical setting in the course over a two-year period (2020 – March 2022) were mapped onto the framework to assess its potential to model solutions stated. Inclusion criteria of project plans were:   1. The plan contains a description of at least one solution which was intended for improving environmental sustainability or climate resilience in a healthcare setting 2. The solution described includes one or more information system components 3. Information can be distilled from the plan about the context of the solution, including the target organization, changes to governance, processes, information exchange, information processing and technical specifications of the solution. 4. The plan was developed in collaboration with healthcare professionals.   Of a total of 42 project plans, 19 were included that met these criteria. |
| Search queries literature review existing frameworks | \| ***Database*** \| ***Query*** \| \| --- \| --- \| \| PUBMED \| ( ("Framework"[Title/Abstract] OR "model"[Title/Abstract] ) AND ("delivery of health care"[MeSH Terms] OR "health facilities"[MeSH Terms] ) AND ("ecological and environmental phenomena"[MeSH Terms] OR "Climate Change"[MeSH Terms] ) AND "Solution"[Title/Abstract] ) \| \| PUBMED \| ("Framework"[Title/Abstract] OR "model"[Title/Abstract]) AND "  Health Information Systems"[MeSH Terms] ) \| \| EBSCO Host \| TI ( framework or model or theory ) AND AB healthcare AND AB environmental impact \| \| EBSCO Host \| TI ( framework or model ) AND AB "health information system" \| |
