# Supplementary material for: Medical informatics and climate change: a framework for modeling green healthcare solutions
Source: J Am Med Inform Assoc. 2022 Oct 11;29(12):2083–8. doi: 10.1093/jamia/ocac182 (PMC9667184; doi:10.1093/jamia/ocac182)
Supplement: ocac182_Supplementary_Data [file ocac182_supplementary_data.zip › ocac182_Supplementary_Data/Supplementary File Appendix B.docx]

Appendix B List with project plans

This table describes the project plans included in this study:

| **Project plan reference** | **Project description** | **Setting/Department** | **Year** |
| --- | --- | --- | --- |
| M1 | Hospital-specific dashboard about waste | Academic hospital | 2020 |
| M2 | Sensors on existing water pipes that record water consumption presented to healthcare professionals in department specific dashboard | Hospital department | 2020 |
| A1 | Marathon-quiz/questionnaire for personnel in App for interested users. Monitors users’ behavior by including questions about behavior, linked to hospital intranet and combined with a hospital specific PowerBI dashboard that presents data monthly about waste. Reward for department with most points obtained. | Academic hospital | 2021 |
| A2 | Mandatory e-learning about waste for personnel | Academic hospital | 2021 |
| I1 | Display greenest medication first in prescription screens of Computerized Physician Order Entry System | Academic hospital | 2021 |
| I2 | Sensors on medication packages to measure shelf-life linked to EHR procurement system, includes facilitating re-use of unopened medication | Pharmacy, Academic hospital | 2022 |
| I3 | Smart pill bottle that replaces sachet packages, logging of medication usage through sensor, interoperability through mobile app to HER portal. Includes push notifications to patients and logging of intake of medication in app. | Patient home, Pharmacy | 2021 |
| I4 | Time till next GP consult affecting the amount of sachet packaged medication ordered by pharmacist, interoperability through EHR | Pharmacy, GP | 2020 |
| I5 | Occupational-driven heating/cooling system combining HVAC system functions with AI prediction models based on hospital schedule and providing input to scheduling (promoting a planning that requires less energy) | Academic hospital | 2022 |
| I6 | Digital scheduling through EHR that promotes treatment of several patients from the same vial at the same time interval (batch scheduling), reducing intravenous medication waste. | Academic hospital | 2021 |
| I7 | Digital prescription system that aids physicians in (green) decision making regarding medication based on LCA data available in EHR | Academic hospital and GP | 2020 |
| I8 | Patient app for planning and tracking medication behavior. Patients are notified when particular medication needs to be taken or when stock is low, incl. patients e-learning on health- and environment-related consequences of medication usage and disposal | Patient home, Hospital | 2021 |
| I9 | Shared inventory system for pharmacies – link inventory systems with AI support to instruct pharmacies to transfer medication to other pharmacies or order new medication. Resulting in smaller inventories, better availability of medication and less medication waste. | Regional pharmacies | 2021 |
| I10 | Replace paper CMIs with a QR codes linking to digital CMIs | Pharmacy, Manufacturing/packaging | 2021 |
| I11 | Digital reminder and awareness distribution tool for the Dutch Cervical Cancer Screening program, reduce paper invitations and reminders by electronic format. Increase attendance rate through improved patient reminding and information (incl. chatbot) | National screening program | 2022 |
| I12 | App for patients with diabetes type 2 and their physicians for patient self-care and communication. Reduce trave. Optimize treatment plans, combine with E-consultation to reduce travel to hospital and GP. Provides functionality to schedule meeting with GP, send report t | Patient home, GP and Academic hospital | 2022 |
| I13 | Food ordering information system with app for admitted patients to promote sustainable food and prevent leftovers. Part of a bigger project that promotes healthy food choices and smaller portions on an increased number of ordering timeslots during the day and night and integrates food ordering process with AI solution predicting food demand based HIS data and bed occupation schedules | Academic hospital and Local food suppliers | 2022 |
| I14 | Platform that helps purchasers to take sustainability criteria into account in the procurement process. On the platform suppliers provide data on (the sustainability of) their practices and products presented by sustainability index (as well as pricing etc.). | Hospital partners and Suppliers | 2020 |
| I15 | Remotely monitor CVD patients to reduce their transportation to hospitals. Patients provided with 4G tablets and wearable monitoring devices. For selected group of patients that is capable of performing self-measurement. Includes remote data management system (receiving patient data, presenting views of data, providing secure access) | Patient home, Academic hospital (coronary care) | 2020 |
