# Supplementary material for: Medical informatics and climate change: a framework for modeling green healthcare solutions
Source: J Am Med Inform Assoc. 2022 Oct 11;29(12):2083–8. doi: 10.1093/jamia/ocac182 (PMC9667184; doi:10.1093/jamia/ocac182)
Supplement: ocac182_Supplementary_Data [file ocac182_supplementary_data.zip › ocac182_Supplementary_Data/Supplementary File Appendix C.docx]

Appendix C Overview of solutions mapped to framework components

| ***Does*** |  | ***Consists of*** |  |  |  | ***Where*** | ***What*** |
| --- | --- | --- | --- | --- | --- | --- | --- |
|  |  |  |  |  |  |  |  |
| **Solution type** | **Summary [projectplan reference Appendix B]** | **Governance architecture** | **Domain Level** | **Logical tool level** | **Physical tool level** | **Healthcare facility** | **Environmental impact** |
| Monitor & assess | Hospital-specific dashboard about waste [M1] | n.a. | Waste management | Dashboard | Desktop app | Hospital | Wastes |
|  |  | Benchmarking | Waste management | Dashboard | AI, desktop app | Hospital | Wastes |
|  | Sensors on existing water pipes that record water consumption [M2] | n.a. | Watermanagement | Dashboard | AI, camera, sensor | Hospital | Water |
| Awareness & knowledge | Marathon-questionnaire for personnel [A1] | n.a. | Staff education | Dashboard, quiz | Email | Hospital | All |
|  |  | n.a. | Staff education | Push notification, quiz | Mobile app, website | Hospital | All |
|  |  | n.a. | Staff education | Dashboard, newspaper, push notification, quiz | Mobile app | Hospital | All |
|  |  | n.a. | Staff education | Campaign, dashboard, newspaper, push notification, quiz | Mobile app, tv screen | Hospital | All |
|  | Mandatory e-learning about waste for personnel [A2] | n.a. | Staff education | Campaign, E-learning | n.s. | Hospital | Wastes |
|  |  | n.a. | Staff education | E-learning | n.s. | Hospital | Wastes |
| Apply intervention | Display greenest medication first (in prescription screens) [I1] | Regulations on scoring environmental impact | Medication prescription | Prescription system | EHR | Hospital | All |
|  | Sensors on medication packages to measure shelf-life [I2] | Medication reuse policy | Medication/product manufacturing, pharmacy operation | Procurement system | EHR, sensor | Hospital, pharmacy | All |
|  |  | Medication reuse policy, medication packaging policy | Pharmacy operation | Visual status | AI, sensor | Pharmacy | Chemicals, wastes |
|  |  | Medication packaging policy, medication reuse policy, | Medication/product manufacturing, pharmacy operation | Inventory system, visual status | Desktop app, sensor | Pharmacy | Chemicals, wastes |
|  | Smart pill bottle that replaces sachet packages  [I3] | Medication packaging policy | Patient self-care, pharmacy operation | Logging, push notification | EHR, interoperability, Mobile app, sensor | Pharmacy | Wastes |
|  |  | Interorganizational agreement, medication packaging policy, | Home care operation, patient self-care, pharmacy operation | Logging, push notification | EHR, interoperability, Mobile app, sensor | Home care, pharmacy | Wastes |
|  | Time till next GP consult affecting the amount of sachet packaged medication ordered by pharmacist [I4] | Medicaiton ordering policy | Pharmacy operation | Prescription system, scheduling system | Desktop app, EHR, interoperability | Home care, pharmacy | Chemicals, wastes |
|  | Occupational-driven heating/cooling system [I5] | n.a. | Building maintenance, workspace allocation | Scheduling system | AI, sensor, thermostat | Hospital | GHGs |
|  | Digital scheduling system that promotes treatment of several patients from the same vial at the same time interval [I6] | n.a. | Patient scheduling | Scheduling system | EHR | Hospital | Chemicals, wastes |
|  | Digital prescription system that aids physicians in (green) decision making regarding medication [I7] | n.a. | Medication prescription | Prescription system | EHR | GP, hospital | Chemicals, wastes |
|  | Patient app for planning and tracking medication behavior [I8] | n.a. | Patient self-care | E-learning, logging, patient portal, patient record, push notification | Interoperability, mobile app | Home care | Chemicals, wastes |
|  |  | E-consultation policy | Patient health monitoring, patient self-care | E-learning, e-consult, logging, patient record, push notification, scheduling system | Email, mobile app | Home care, GP, hospital | Chemicals, wastes |
|  | Shared inventory system for pharmacies [I9] | Interorganizational agreement | Pharmacy operation | Inventory system | AI, cloud, desktop app, interoperability | Pharmacy | Chemicals, wastes |
|  | Replace paper CMIs with a QR codes linking to digital CMIs [I10] | National GDP guideline | Medication/product manufacturing, patient self-care, pharmacy operation | Barcode | Website | Pharmacy | GHGs, wastes |
|  |  | National GDP guideline | Medication/product manufacturing, pharmacy operation | Barcode, patient portal, patient record | Desktop app, website | Pharmacy | GHGs, wastes |
|  | Digital reminder and awareness distribution tool for the Dutch Cervical Cancer Screening program [I11] | National protocol, interorganizational agreement | Screening program operation | Campaign, push notification | Email, interoperability, mobile app, website | Public health institute | All |
|  |  | National protocol | Patient scheduling, screening program operation | Barcode, campaign, patient portal, push notification, scheduling system | Email, interoperability, mobile app, website | Public health institute | All |
|  |  | Interorganizational agreement, national protocol, | Patient scheduling, screening program operation | Barcode, campaign, patient portal, push notification, scheduling system, chatbot | AI, email, interoperability, mobile app, website | Public health institute | All |
|  | App for patients with diabetes type 2 and their physicians for patient self-care and communication [I12] | E-consultation policy | Patient health monitoring, patient self-care | e-consult, health planning and tracking, patient record | Desktop app, EHR, interoperability, mobile app | Home care, hospital, GP | All |
|  |  | E-consultation policy | Patient health monitoring, patient self-care | E-consult, health planning and tracking, logging, patient record | Desktop app, EHR, interoperability, mobile app, sensor | Home care, hospital, GP | All |
|  | Food ordering app for admitted patients to promote sustainable food and prevent leftovers [I13] | n.a. | Food service | E-learning, procurement system | Mobile app, tablet | Hospital | All |
|  |  | n.a. | Food service | E-learning, procurement system | AI, mobile app, tablet | Hospital | All |
|  |  | Interorganizational agreement | Food service | E-learning, procurement system | AI, interoperability, mobile app, tablet | Hospital | All |
|  |  | n.a. | Food service | E-learning, procurement system | Desktop app, interoperability, mobile app, tablet | Hospital | All |
|  | Platform that helps purchasers to take sustainability criteria into account in the procurement process [I14] | Regulation on scoring environmental impact | Procurement | Procurement system | Desktop app | Hospital | All |
|  |  | Regulation on scoring environmental impact | Procurement | Dashboard, procurement system | Website | Hospital | All |
|  | Remotely monitor CVD patients [I15] | n.a. | Patient health monitoring, patient self-care | Logging, patient record | Desktop app, interoperability, mobile app, sensor, tablet | Hospital, home care | all |

AI: artificial intelligence; app: application; CMI: consumer medication information; CVD: cardiovascular disease; GDP: good distribution practice; GP: general practitioner; n.a.: not applicable; n.s.: not specified; QR: quick response.
